# Development of personas to tailor prehabilitation care for patients undergoing cancer surgery: An exploratory study

**DOI:** 10.1016/j.invent.2026.100954

**Published:** 2026-05-20

**Authors:** Karin Valkenet, Miriam van der Velde, Petra Bor, Edwin Geleijn, Willem M. Otte, Germijn Heijnen, Ad Kerst, Anne-Marie Pierik-van Roest, Rik Bijl, Marije Marsman, Marike van der Leeden

**Affiliations:** a Department of Rehabilitation, Physiotherapy Science and Sport, University Medical Center Utrecht, Utrecht University, Utrecht, the Netherlands; b Research group Innovation of Human Movement Care, HU University of Applied Sciences Utrecht, Utrecht, the Netherlands; c Department of Rehabilitation Medicine, Amsterdam University Medical Center, Vrije Universiteit Amsterdam, Amsterdam, the Netherlands; d Department of Child Neurology, University Medical Center Utrecht, Utrecht University, Utrecht, the Netherlands; e Department of Oncological Surgery, University Medical Center Utrecht, Utrecht University, Utrecht, the Netherlands; f Department of Anesthesiology, Amsterdam University Medical Center, Vrije Universiteit Amsterdam, Amsterdam, the Netherlands; g Department of Anesthesiology, University Medical Center Utrecht, Utrecht University, Utrecht, the Netherlands

**Keywords:** Personas, Prehabilitation, Oncology, Personalized care, Tailored care, Healthcare

## Abstract

**Background:**

Prehabilitation is increasingly used to improve postoperative outcomes. Despite the growing emphasis on tailored care, healthcare nowadays is often highly protocolized. Personas, fictional but realistic representations of patients and their needs, might be helpful to design and deliver tailored prehabilitation support.

**Methods:**

Exploratory multi-methods study. First, the Nominal Group Technique was used to establish a list of patient characteristics for determining the appropriate mode of prehabilitation support. Second, a cluster analysis was performed and the Proto Persona Development Method was used to develop personas. Third, the modes of prehabilitation support deemed appropriate were determined per persona. Fourth, a patient consultation session was conducted.

**Results:**

The first step resulted in a list of 29 patient characteristics: physical fitness, health literacy, frailty, personal preference and motivation were ranked as most important for tailoring prehabilitation. The cluster analysis (*n* = 291) did not result in distinctive clusters. With experts, four prehabilitation personas were developed: Avoiding Alex, Informed Indy, Overwhelmed Ollie and Social Sam. The interrater reliability of patient characteristics and modes of support per persona ranged from moderate to excellent.

**Conclusions:**

The personas show the importance of providing different communication and modes of prehabilitation care. Their use might avoid cognitive biases by adapting the information to the needs per individual patient.

## Highlights

• Despite the growing emphasis on tailored care, healthcare nowadays is often highly protocolized.
• Prehabilitation can be delivered as supervised therapy, as hybrid care and as fully digital guidance.
• Personas, fictional but realistic representations of patients, might be helpful to tailor prehabilitation support.
• With experts, four prehabilitation personas were developed: Avoiding Alex, Informed Indy, Overwhelmed Ollie and Social Sam.
• The personas show the importance of providing different communication and modes of prehabilitation care.

## 1 Introduction

In the last 25 years, the incidence of new cancer diagnoses has doubled resulting in an increasing number of patients undergoing elective surgery for cancer (Nederland IK, 2023; Bray et al., 2024; Xiang et al., 2022). Among these patients, a subset faces an increased risk of complications due to a low physical fitness level and life style risk factors prior to the surgery (Tew et al., 2018; Argillander et al., 2022; Steffens et al., 2021; West et al., 2016). Consequently, this group has higher postoperative healthcare consumption leading to additional healthcare costs (Sabajo et al., 2024; Loor et al., 2021).

To decrease postoperative complications and thereby decrease healthcare costs, prehabilitation has gained increasing attention in the last decade (Sabajo et al., 2024; Skorepa et al., 2024). Prehabilitation consists of physical and mental interventions before surgery to increase a patients' resilience to better withstand the upcoming stressor of the surgery (Topp et al., 2002; Scheede-Bergdahl et al., 2019). As patients with cancer represent a diverse population, each with unique physical, psychological and social challenges, prehabilitation must be tailored to individual preferences and needs of patients to maximize its impact. In addition, to maintain the affordability and accessibility of healthcare and prehabilitation, it is important to deliver the right care in the right place.

Nowadays there are multiple modes of prehabilitation available. Prehabilitation can be delivered as supervised therapy (Weston et al., 2016), as hybrid care (Moyen et al., 2025) and as fully digital guidance (van der Velde et al., 2025; van der Velde et al., 2021). Despite the growing emphasis on tailored care, healthcare nowadays is often highly protocolized and healthcare professionals often lack practical instruments to effectively determine the most appropriate mode of delivery per patient and to address the varying patient preferences and needs.

To better understand the preferences and needs of patients, the Capability, Opportunity, Motivation, Behavior [COM—B] model is a useful framework (van der Velde et al., 2023). This model, part of the Behavior Change Wheel, offers a practical framework to understand and influence patient behavior (Michie et al., 2011). Participation in prehabilitation is shaped by factors across all COM-B components, highlighting the importance of addressing capability, opportunity and motivation (van der Velde et al., 2023).

To help healthcare providers in designing and tailoring prehabilitation, the use of personas might be helpful. Personas are fictional but realistic representations of user groups of a product or service (Bartels et al., 2023). The development of personas is an often used method in user-centered design as aid to understand characteristics, goals, needs and behaviors of users (LeRouge et al., 2013). Using personas in the early phases of the design process can help identify product features and demonstrate how these features address user needs and challenges, leading to the development of successful products (Varzgani, 2023).

In healthcare, cognitive biases may impact clinical practice by providing information according to the preferences of the healthcare practitioner, instead of adapting the information to the needs of the patient in front of them (Ennis and Vapiwala, 2021). Personas can be used to communicate health information through relatable first-person narratives. Information delivered in this form, instead of a list of facts, can improve attitudes and beliefs about others and motivate prosocial behavior (Massey et al., 2021; Andrews et al., 2022).

Therefore, the purpose of this study was to create personas to support healthcare practitioners in designing and delivering tailored prehabilitation before cancer surgery. During the development of the personas the COM-B model and the Proto Persona Development Method were used (Michie et al., 2011; Varzgani, 2023).

## 2 Methods

### 2.1 Study design

This was an exploratory multi method study using qualitative and quantitative methods performed between November 2022 and July 2024. A sequential design was used where one method was used first and its findings shaped the next step (Creswell, 2018). First, a focus group was organized to identify the patient characteristics that healthcare practitioners believed of influence on the appropriate level and mode of prehabilitation support. Second, prehabilitation personas were developed using a cluster analysis on pooled quantitative studies. Posthoc, as the cluster analysis did not yield distinct clusters, subject matter expert meetings were conducted to develop proto personas as an alternative approach. This additional step was not planned a priori, but was deemed necessary to achieve the study objectives in the absence of clear quantitative clustering. Third, an expert panel determined which patient characteristics and mode of support per persona they deemed appropriate. Fourth and final, a patient consultation session was conducted to gather preliminary input about their thoughts on the developed personas and the usability in daily care.

Participants of the pooled quantitative studies gave written permission for the use of their data in future research. The studies were approved by local medical ethics committees of Amsterdam University Medical Center and University Medical Center Utrecht (study protocol numbers NL61503.029.18 and 19-026 respectively) (van der Velde et al., 2025; Bor et al., 2022).

### 2.2 Step 1. Identification of relevant patient characteristics

A focus group was organized including healthcare professionals from different disciplines (nurse practitioners, physiotherapists, researchers, physicians) and organizations (academic and top clinical hospitals in the Netherlands). The Nominal Group Technique (NGT) was used to achieve consensus on relevant patient characteristics. With NGT, idea generation and problem solving are combined in a structured group process (Gallagher et al., 1993; Van de Ven and Delbecq, 1972). First, participants were asked to write down which patient characteristics they perceived to be of influence on the suitable mode of support (e.g. supervised, hybrid, digital) for patients in the preoperative phase. Subsequently, participants were invited one by one to present their most important characteristic. This characteristic was written down by the moderator of the session without discussion. When a characteristic was already mentioned by a fellow-participant, the participant presented their second most important characteristic. This was repeated until participants presented all the characteristics. Following, all characteristics were ranked per participant by allocating points to five characteristics: the most important characteristic received five points, the least important one received one point. The NGT meeting was organized as a video conference, and the digital canvas tool Miro (San Francisco, USA) was used for reporting and ranking the characteristics.

After the NGT meeting, the project team (KV, MvdL, EG, MV and PB) individually allocated the characteristics to the six categories of the COM-B model: physical capability, psychological capability, physical opportunity, social opportunity, reflective motivation, automatic motivation (van der Velde et al., 2023; Michie et al., 2011). Following, a consensus meeting was organized with the project team to reach consensus of the final allocation of the variables across the COM-B categories.

### 2.3 Step 2A. Persona development using cluster analysis

By grouping similar objects into clusters, cluster analysis helps uncover patterns and structures that might not be immediately apparent. It aims to achieve the highest homogeneity within a cluster, as well as the lowest homogeneity between clusters (van der Esch et al., 2015). In order to develop a database with a wide range of variables for the cluster analysis, existing datasets were sought by contacting experts on prehabilitation in the Netherlands. Datasets were included if they (1) investigated patients prior to oncological surgery and (2) included baseline variables from at least one other COM-B category than physical capability and physical opportunity to ensure enough variation in data variables. The enquiry yielded that the majority of studies only included variables on the physical capability category of COM-B and were therefore ineligible for inclusion in this study.

Finally, data of the two following studies were used for the cluster analysis:

1. **“**Be Prepared”: A multicenter randomized controlled trial (RCT) examining the effects of a mobile application that provides patients with advice on risk behaviors before and after their surgery (van der Velde et al., 2025). The RCT included patients undergoing major surgery having at least one risk behavior (currently smoking, ≥7 alcohol consumptions a week, moderate-intensity physical activity <30 min every day, muscle-strengthening activities on <2 days a week, and unintentional weight loss of >3 kg in the last month) (van der Velde et al., 2025). Of the total sample, only patients undergoing oncological surgery were included in the cluster analysis.

2. **“**PAM-ONCO”: A monocenter observational study examining the course of physical functioning and mental functioning in patients undergoing major cancer surgery of the esophagus, liver, colon, stomach, bladder or pancreas (Bor et al., 2022). All patients were included in the cluster analysis.

The variables identified in step 1 were used as clustering variables when available in both datasets. When variables of the same construct were measured with different outcome measures, the outcomes were standardized to a single scale. Afterwards, the datasets were merged into one pooled dataset. Unsupervised clustering was performed on the preoperative data to identify latent patterns and hidden structures in our sample (Eckhardt et al., 2023). The Gower distance (scaled to a 0 to 1 range) was calculated were zero corresponds with identity and one with a maximal dissimilarity (Gower, 1971). The optimal number of clusters (k = 2–10) was determined using the iterative clustering procedure Partitioning Around Medoids (PAM) in combination with the silhouette width to assess the quality of a cluster (Milligan and Cooper, 1985). The silhouette width can range from −1 to 1, where higher values correspond with better separation (Rousseeuw, 1987). The minimal adequacy threshold for silhouette width was set at 0.5 (Hubert LJaA, 1985).

### 2.4 Step 2B. Persona development with subject matter experts

Because the cluster analysis did not yield clusters with a silhouette width of 0.5 or higher, posthoc it was decided to develop proto personas with subject matter experts. Proto personas are assumption-based personas that are created through indirect interaction with the users (Varzgani, 2023). The Proto Persona Development Method was used as guideline which describes three steps: (1) identification of key user attributes, (2) user data collection from subject matter experts, and (3) analysis of the collected data and creation of proto personas (Varzgani, 2023). These steps align with our steps 1, 2B and 3.

A subject matter expert meeting was organized to develop prehabilitation proto personas. To collect user data from experts, a persona worksheet was developed where characteristics, goals, needs and behaviors of fictional but realistic patients could be recorded (Appendix). In the expert meeting, different healthcare practitioners (nurse practitioners, dieticians, physiotherapists and physicians) were asked to imagine ‘typical patients’ and to fill out one persona worksheet per typical patient. The experts could complete as many persona worksheets as they could in 45 min. Following, the experts presented their persona worksheets to each other and grouped them when the worksheets had overlapping descriptions.

Afterwards, project group members (KV, EG, MvdV) analyzed the collected data by discussing similarities and discrepancies in the recorded characteristics both within and between the groups of worksheets. Final personas were established by adhering as closely as possible to the groups and characteristics identified by the experts. A member check was conducted to verify whether the experts recognized their persona worksheets in the final personas.

### 2.5 Step 3. Scoring patient characteristics and prehabilitation support per persona

Subject matter experts were asked individually to score all patient characteristics from step 1 per persona. They were asked to score to what extent they perceived the characteristics to be present in the persona on a scale from 1 (very low) to 5 (very high). In addition, the experts scored their perceived suitability of different kinds of prehabilitation support (individual face to face sessions, video consulting, mHealth/eHealth, group sessions, guidance by family/friends, peer support) per persona on a scale from 1 (not suitable) to 5 (very suitable). To evaluate the level of agreement between experts, Intraclass Correlation Coefficients were calculated per persona using a two-way random consistency model (SPSS version 31.0.0.0). Values less than 0.5, between 0.5 and 0.75, between 0.75 and 0.9, and greater than 0.90 are classified as poor, moderate, good, and excellent interrater reliability, respectively (Koo and Li, 2016).

### 2.6 Step 4. Patient consultation

Finally we performed a patient consultation step in a 1.5 h group session after the development of the personas. Participants were explained our idea to use personas to tailor care and how the personas were established. Three open ended questions were asked and participants were invited to write down their ideas on sticky notes, which were discussed thereafter. Participants were asked 1) to share their first thoughts about the personas, 2) whether they recognized themselves in one or more of the personas and what their thoughts were, and 3) their ideas about how the personas could be used in daily care.

## 3 Results

### 3.1 Step 1. Identification of relevant patient characteristics

In total nine prehabilitation experts from four different hospitals (academic and top clinical) participated in the NGT meeting: two nurse practitioners, two physiotherapists, two researchers, one surgeon, one anesthetist and one rehabilitation physician assistant. The meeting was moderated by two researchers (KV and MvdV).

After the first NGT round, 29 patient characteristics were identified by the experts to be of influence on the suitable mode of prehabilitation care. After the round of ranking, 22 of the characteristics received points (range 1 to 33) and seven did not receive points (Table 1).

**Table 1:** List of patient characteristics identified in the NGT meeting and used in the cluster analysis, grouped according to the COM-B model.

*NGT score*	*NGT variables*	*Standardized cluster variables*
	Physical capability	Physical capability
33	Current level of functioning and activities	30 min moderate/vigorous exercise 5 days a week (yes/no) Muscle strengthening activities 2 days a week (yes/no) Physical functioning (0–100)^a^
7	Nutritional status	
4	Prognosis (what can be reached with prehabilitation)	
2	Comorbidities	ASA classification (1–4)^b^
2	Experience with exercise or sports	
0	Current acute complaints (physical, mental)	Fatigue (0–10)^c^

	Physical opportunity	Physical opportunity
4	Financial situation	
0	Living situation	Living alone (yes/no)
0	Ability to travel independently	
0	Complexity of the surgery	
0	Time span until surgery	

	Social opportunity	Social opportunity
4	Social network	
3	Opinion of social environment	
2	Culture	

	Psychological capability	Psychological capability
18	Health literacy	
6	Cognition/ Mental competency	Level of education (3 categories)^d^
4	Control and acceptation of health status	Control (Likert 1–7)^e^ Acceptation (Likert 1–7)^e^
4	Digital skills	
3	Coping style	
3	Self-management skills	
2	Illness perception	
0	Language skills	

	Reflective motivation	Reflective motivation
13	Personal preference (for guidance)	
8	Motivation	
8	Self-effectiveness	Self-effectiveness (0–10)^f^
1	Personal goal(s)	
0	Need for structure	

	Automatic motivation	Automatic motivation
3	Anxiety and depression	Anxiety and depression (1–3)^g^

	No Category	
16	Resilience	

The clustering variables were measured with the following instruments in the BePrepared and the PAM-ONCO study respectively: ^a^CAT PROMIS Physical Functioning (Abma et al., 2021) and AMPAC outpatient (Thackeray et al., 2021) ^b^American Society of Anesthesiologists classification (Brouquet et al., 2010) ^c^Global Fatigue score and Short Fatigue Questionnaire (Alberts et al., 1997) ^d^primary education only, secondary education, higher eduction ^e^Subjective Believed Health questionnaire (Bloem and Stalpers, 2016; Bor et al., 2024) ^f^General self-efficacy scale (Scherbaum et al., 2006) ^g^EQ5D3L anxiety/depression question (Rabin and de Charro, 2001) resp. HADS (Lemay et al., 2019).

After the consensus meeting of the project team members (KV, MvdL, EG, MvdV, PB) 28 of the 29 variables were allocated across the COM-B categories (Table 1, left column). Resilience was considered as an overarching construct and was therefore not allocated.

### 3.2 Step 2A. Persona development using cluster analyses

From the Be Prepared dataset, 193 participants undergoing oncological surgery were included from University Medical Center Utrecht (*n* = 99) and Amsterdam University Medical Center, location Vrije Universiteit medical center (*n* = 94). Data from the PAM-ONCO dataset resulted in an additional 98 participants from University Medical Center Utrecht.

This resulted in a total sample of 291 participants undergoing surgery for cancer in the pooled dataset (Appendix). After matching the patient characteristics from step 1 to the available variables in the pooled dataset, 11 clustering variables were identified (Table 1, right column). In addition, age (continuous) and gender (male/female) were included.

The cluster analysis showed the highest silhouette width value of 0.105 with k = 7 clusters. As the threshold of 0.5 was not reached, these results were considered insufficient for the development of personas.

### 3.3 Step 2B. Persona development with subject matter experts

**Fig. 1:**
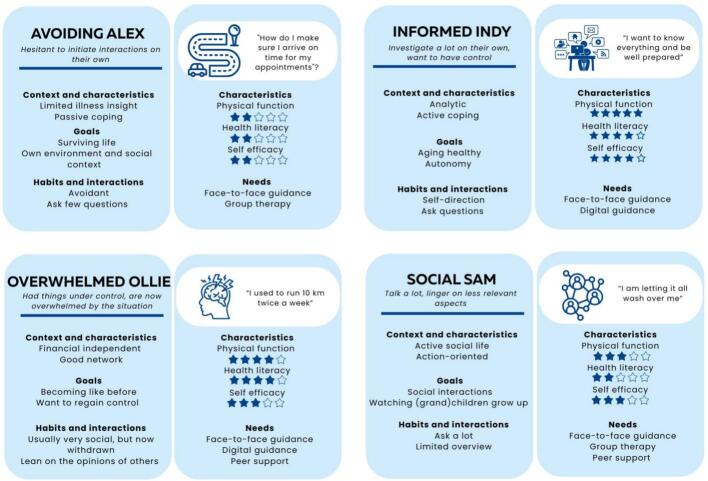
Visual presentation of the first concept of proto personas developed by subject matter experts.

As the cluster analysis did not result in distinctive clusters, prehabilitation personas were developed with subject matter experts. In total five experts (two physiotherapists, one anesthetist, one nurse practitioner and one physician assistant rehabilitation) joined this expert meeting. In total, the experts filled out 15 different persona worksheets. After discussion, 12 of the sheets were matched into four groups with similar characteristics and needs. Three persona worksheets remained unused as they could not be matched into one of the four groups but were also found unsuitable to form a separate group. Of the unmatched persona worksheets one was called ‘the optimist’ always seeing opportunities, another ‘the pessimist’ with doubts about the treatment and dreading the situation, and the final ‘the co-morbid patient’ who is used to living with disabilities. Based on the grouped persona worksheets, four gender-neutral personas were composed: Avoiding Alex, Informed Indy, Overwhelmed Ollie and Social Sam. After a member check, the personas were finalized (Fig. 1).

**Avoiding Alex** is anxious to initiate interaction with healthcare providers. Alex has low health literacy skills and a passive coping style.

“How do I make sure I arrive on time for my appointments and arrange transportation?”

Has trouble reproducing what was said at the hospital. For Alex life is about surviving and worrying about how to pay the rent and cigarettes, and about taking care of the dogs and making sure that they get their daily walk. Alex is easily worried, is afraid of the unknown and has financial issues. Spends a lot of time at the couch. Has limited health literacy skills, does not use technology a lot but maintains contact with family and friends via mobile text messaging.

**Informed Indy** is looking for ways to get control over the situation and is proactive in finding information online. Indy is motivated, feels in control, likes technology and has a healthy lifestyle.

“I want to know everything and be well prepared for the surgery and the period afterwards”.

Has an active coping style, asks a lot of questions, is motivated. Has always worked in an analytical job and is financially independent. Indy is motivated by healthy aging, autonomy and getting back to normal. Does not believe everything the healthcare practitioner says. Does not like uncertainty, is afraid of getting dependent of others and has the tendency to arrange everything compulsively prior to the surgery. Has good digital skills.

**Overwhelmed Ollie** had everything well under control before the cancer diagnosis. Ollie is overwhelmed and shaken by the situation; never expected to have to go through this.

“I used to run 10km twice a week”

Had a managerial position and was doing okay financially and socially. Is now depressed and has trouble getting into action. Finds it hard to look further than the current misery and is afraid that it will never be the same. Feels lost. Ollie was socially active before the diagnosis, but has the tendency to withdraw in periods of stress. Has good digital skills, but finds technology not very important. Leans on the opinion of others.

**Social Sam** talks a lot and lingers on aspects that are less relevant. Has an active social life. Fears being alone and not being able to understand medical advice.

“I'm letting it all wash over me”

Family and friends are very important for Sam. Likes everything as it is and has trouble with changes. Has a practical job, asks a lot but has limited overview. Lives in a village and feels responsible for the community. Getting back home is the main motivation for Sam, gets home sick quickly. Is afraid of the surgery, is afraid to die. Is also afraid of being alone, and having to eat and drink things that he/she is not used to.

### 3.4 Step 3. Scoring patient characteristics and prehabilitation support per persona

Per persona, the median (min-max) scores from the experts of the top 6 patient characteristics and type of guidance are displayed in Table 2. The Intraclass Correlation Coefficients (ICCs) (95% CI) including all patient characteristics from step 1, were as following: Avoiding Alex 0.85 (0.71–0.93), Informed Indy 0.79 (0.61–0.91), Overwhelmed Ollie 0.73 (0.49–0.87) and Social Sam 0.74 (0.51–0.88). The ICCs (95% CI) of the modes of prehabilitation support per persona were: Avoiding Alex 0.84 (0.48–0.98), Informed Indy 0.84 (0.46–0.97), Overwhelmed Ollie 0.74 (0.16–0.96) and Social Sam 0.94 (0.82–0.99). The ICCs indicate moderate to excellent agreement between experts for both the patient characteristics and the modes of support.

**Table 2:** Medians (range⁎) of expert scores per persona.

	Avoiding Alex	Informed Indy	Overwhelmed Ollie	Social Sam
Patient characteristic⁎⁎ (Top 6)				
Current level of function and activities	2 (1–3)	5 (4–5)	4 (4–5)	3 (2–4)
Health literacy	2 (1–2)	4 (4–5)	4 (4–5)	2 (2–3)
Frailty	4 (3–5)	3 (1–3)	4 (1–4)	3 (3–4)
Personal preference	3 (2–3)	5 (4–5)	4 (3–5)	4 (3–4)
Motivation	2 (2–3)	4 (4–5)	4 (2–5)	3 (3–4)
Self-efficacy	2 (1–2)	4 (4–5)	4 (2–4)	3 (2–3)

Mode of prehabilitation care⁎				
Face to face	5 (1–5)	4 (3–5)	4 (4–5)	5 (5–5)
Digital guidance via screen	2 (1–3)	4 (4–5)	5 (4–5)	3 (2–4)
Digital guidance via mHealth/eHealth	1 (1–2)	4 (3–5)	4 (2–5)	2 (1–3)
Group session supervised by HCP	4 (4–5)	2 (2–3)	3 (1–5)	5 (4–5)
Guidance by family & friends	3 (2–4)	3 (2–4)	3 (2–4)	4 (1–4)
Peer support	3 (2–5)	3 (2–5)	4 (1–4)	5 (4–5)

*HCP* *= Healthcare practitioner.*

⁎ Range of scores by the subject matter experts (min-max).

⁎⁎ *Scored on a Likert scale from 1 to 5.*

The results show that the persona Avoiding Alex scores relatively low on current level of function and activities, health literacy skills, motivation and self-efficacy. For Alex face to face supervision and group sessions were deemed the most suitable. The personas of Informed Indy and Overwhelmed Ollie score high on current level of function and activities, health literacy skills and personal preference. Individual face to face supervision and digital guidance were found suitable for them, as well as peer support for Ollie as this persona also scored high on frailty. The persona of Social Sam scored neutral on characteristics like current level of function and activities, motivation and self-efficacy and low on health literacy. Face to face supervision, group sessions and peer support scored the highest for this persona.

### 3.5 Step 4. Patient consultation

Three patients (two male, one female) who underwent cancer surgery (esophageal cancer 15 years ago, esophageal-cardia cancer 3 years ago, colon cancer 2 years ago) participated in the group session. The participants were enthusiastic about the personas as a tool to tailor care and facilitator of the right care. Two participants recognized themselves in one of the personas (Social Sam and Informed Indy respectively), the third participant could not classify oneself as it differed per moment in time. The participants recognized all the fears and frustrations formulated across the personas and mentioned that they had experienced them all throughout their patient journey.

They stated that it can be dangerous to classify people into categories. They suggested that the personas should be used to support discussion about different types of care. They also questioned whether healthcare professionals are able to make a correct appraisal as they only have limited time per patient. Furthermore, there was discussion about the names of the personas, as some were considered judgmental. One participant suggested to use more neutral names or colors were another found the current names helpful.

## 4 Discussion

Our study resulted in a comprehensive set of patient characteristics that are deemed of influence on the suitable mode of prehabilitation support per patient. As unsupervised clustering lacked separability, four proto personas were developed with subject matter experts: Avoiding Alex, Informed Indy, Overwhelmed Ollie and Social Sam. A description of their different contexts, habits, motivations and fears, and the modes of support deemed appropriate was determined by experts. The distinct differences between the personas show the importance of designing and tailoring prehabilitation support to the unique experiences, challenges and needs of patients awaiting major cancer surgery.

The first step of our study resulted in a rich overview of patient characteristics that were perceived of influence on the mode of support for patients in the overwhelming period before cancer surgery. The identified patient characteristics were divided across all six categories of the COM-B model which indicates the importance of gathering a wide range of patient information to enable personalized care. Unfortunately, our results also show that the majority of prehabilitation studies in the Netherlands only collected variables in one of the COM-B categories, namely the category physical capability. Recent systematic reviews including studies across the world show a similar tendency with the majority of studies focusing on functional outcomes like muscle strength or physical activity (Skorepa et al., 2024; Alsuwaylihi et al., 2024). Although health literacy and personal preference were ranked as two of the most important variables of influence on the right prehabilitation support, they are not recorded in prehabilitation studies. The same applies for mental aspects: only a minority of studies included psychological measurements like anxiety, depression and self-efficacy (Skorepa et al., 2024; Alsuwaylihi et al., 2024). While prehabilitation is multimodal with psychological support as important component, research approaches the patient and prehabilitation in a predominant unimodal way. This shows that a one-sided view of patients undergoing cancer surgery is created in current research and that more comprehensive data collection is needed in order to be able to tailor care to a patient's needs.

In addition, only 11 of the 29 patient characteristics identified in step 1 could be matched to available variables in the pooled dataset. This constrained the cluster analysis as the majority and the most important variables identified by experts were missing.

In the subsequent steps we developed four proto personas. Personas can help to increase awareness in healthcare practitioners on the different patient needs for guidance in the preoperative phase. The personas can be used to communicate health information through relatable first-person narratives, instead of providing information according the personal preferences of the healthcare practitioner. Furthermore, the personas can be used to design comprehensive prehabilitation interventions. In prehabilitation, protocols typically offer either supervised physiotherapy or none at all, with little room for variation based on patient characteristics, needs or preferences. Offering different options to a patient and determining together which type of guidance is most appropriate might result in higher adherence as shared decision making is known for increased feeling of ownership in patients (Bauer and Chiappelli, 2010). Additionally, the personas can be used to tailor care by stratification (Dekkers and Hertroijs, 2018). Our personas show that some patients (like Informed Indy) can be helped with mainly digital support while for others (patients like Social Sam) group sessions and peer support are more suitable. Therefore, the personas can be used to determine specific design features that should be incorporated in a prehabilitation platform to accommodate both Avoiding Alex, who has limited digital skills and low health literacy, and Informed Indy, who is tech-savvy and proactive. In addition, stratification can help to keep prehabilitation care affordable, as not every patient will need face to face supervision. However, there is a risk that stratification is used as a simple flow chart, leading to labeling people based on presumptions and without involving the patient. Furthermore, although the personas are presented as recognizable groups, there is diversity within each persona. People might identify themselves with multiple personas. Therefore, it is essential to incorporate shared decision making into this approach and to use it as a conversation tool.

Although our study focused on the use of personas in the preoperative phase, personas can be used throughout the entire patient journey. In a study of Bor et al. (2024) patients undergoing major oncological surgery were allocated into four patient profiles based on their level of acceptance (high/low) and perceived control scores (high/low) at different moments in their patient journey. Interestingly, some patients changed from patient profile over time. The majority of patients who were preoperatively classified into the profile with high acceptance and high perceived control, changed to one of the other profiles with low acceptance and/or low perceived control after discharge. At the same time, patients who were preoperatively classified into the profile with low acceptance and low perceived control tended to remain in this profile after discharge. This underlines the necessity to keep tailoring care across time and to reevaluate the needs and wishes of patients multiple times during their patient journey.

A strength of our study is that we transparently describe our research process with both planned and unplanned methodologies. We deliberately choose to present the insufficient results of the cluster analysis and our pivot to the proto persona approach with subject matter experts. By doing that, we were transparent about our alternative analytic paths and refrained from selective reporting of findings. Another strength is that we have included multiple experts from different disciplines and organizations in the Netherlands in order to capture the perspectives of experienced healthcare practitioners treating a wide range of patients. Our results might therefore even be applicable to other patient groups as the patient variables used to develop the personas are not specifically associated with cancer surgery. However, inclusion of a bigger panel of healthcare professionals and multiple perspectives of each profession would have enriched our results and increased the generalizability. Organizations interested in using these personas should therefore first determine whether the personas are recognized by healthcare practitioners and patients within their organization before adopting them in designing and delivering prehabilitation.

Although we have performed a group session to gather preliminary input from patients on the developed personas, a major limitation of our study is that we did not include patients from the beginning in our expert meetings. Patient participation is righteously encouraged to enhance the development of healthcare interventions that align with patients' needs. Therefore, the next step should be to validate the personas with both qualitative and quantitative patient data and to adjust the personas accordingly, and investigate the alignment of persona allocation between patients and healthcare professionals. Another limitation is the small sample we used in the cluster analysis. Although no clear sample size guidelines exist for cluster analyses, sample sizes of at least 70 times the number of variables are suggested (Dolnicar et al., 2013). Although other studies used lower sample sizes successfully, it is plausible that our sample size was too small for this data-driven approach.

We conclude that in daily care and in future research it is important to assess a wide range of patient characteristics in different domains (e.g. physical, psychological, motivation, context) in order to enable tailored care. Personas can be a promising tool for supporting healthcare practitioners in designing and tailoring care to the individual patient and can be valuable to increase awareness of the need to offer multiple modes of support and guidance. Future research should explore patients' perceptions of persona use in order to further develop the presented personas, examine the support required by healthcare practitioners to effectively apply personas in delivering tailored care, and evaluate the impact of personas on prehabilitation outcomes and compliance to prehabilitation programs.

## Credit authorship contribution statement

KV designed the methods, performed the data collection and the analyses of step 1, 2B and 3, and wrote the manuscript, MV, PB, EG and ML helped with the design, data collection and gave feedback on the manuscript, WMO performed the analyses of step 2A and gave feedback on the manuscript, GH, AK, AP, RB and MM helped with the data collection and gave feedback on the manuscript.

## Funding information

This study was funded by National Fund Against Cancer and Foundation Fit4Surgery.

## Declaration of competing interest

The authors declare no conflict of interest.

## Data Availability

Collected data will be shared upon reasonable request.
