# Supplementary material for: Development of personas to tailor prehabilitation care for patients undergoing cancer surgery: An exploratory study
Source: Internet Interv. 2026 May 20;44:100954. doi: 10.1016/j.invent.2026.100954 (PMC13217499; doi:10.1016/j.invent.2026.100954)

**APPENDIX**

*Characteristics of included participants (n=291) in the clustering analyses (step 2A)*

| Age, mean (SD) | 63.0 (10.8) |
| --- | --- |
| Male gender, n (%) | 166 (57) |
| BMI, mean (SD) | 26.3 (5.1) |
| Physical Functioning (0-100), mean (SD) | 52.8 (11.6) |
| Fatigue (0-10), mean (SD) | 6.5 (2.1) |
| Self-efficacy, mean (SD) | 7.8 (1.5) |
| Higher education | 103 (39.5) |
| Living alone | 54 (20.1) |
| Anxiety and Depression |  |
| None | 181 (63.5) |
| Moderate | 90 (31.6) |
| Severe | 14 (4.9) |
| ASA classification |  |
| I | 27 (9.5) |
| II | 160 (56.3) |
| III | 90 (31.7) |
| IV | 7 (2.5) |
| Preference for digital guidance | 148 (51.0) |
| Surgical specialty |  |
| Gastrointestinal | 202 (69.4) |
| Cardiothoracic | 33 (11.3) |
| Urology and Gynecology | 32 (11.0) |
| Head and Neck | 16 (5.5) |
| Other | 8 (2.8) |
| Complication after surgery in hospital | 106 (35.8) |
| LOS, mean (SD) | 8.5 (7.7) |

**APPENDIX**

Persona worksheets (step 2B)


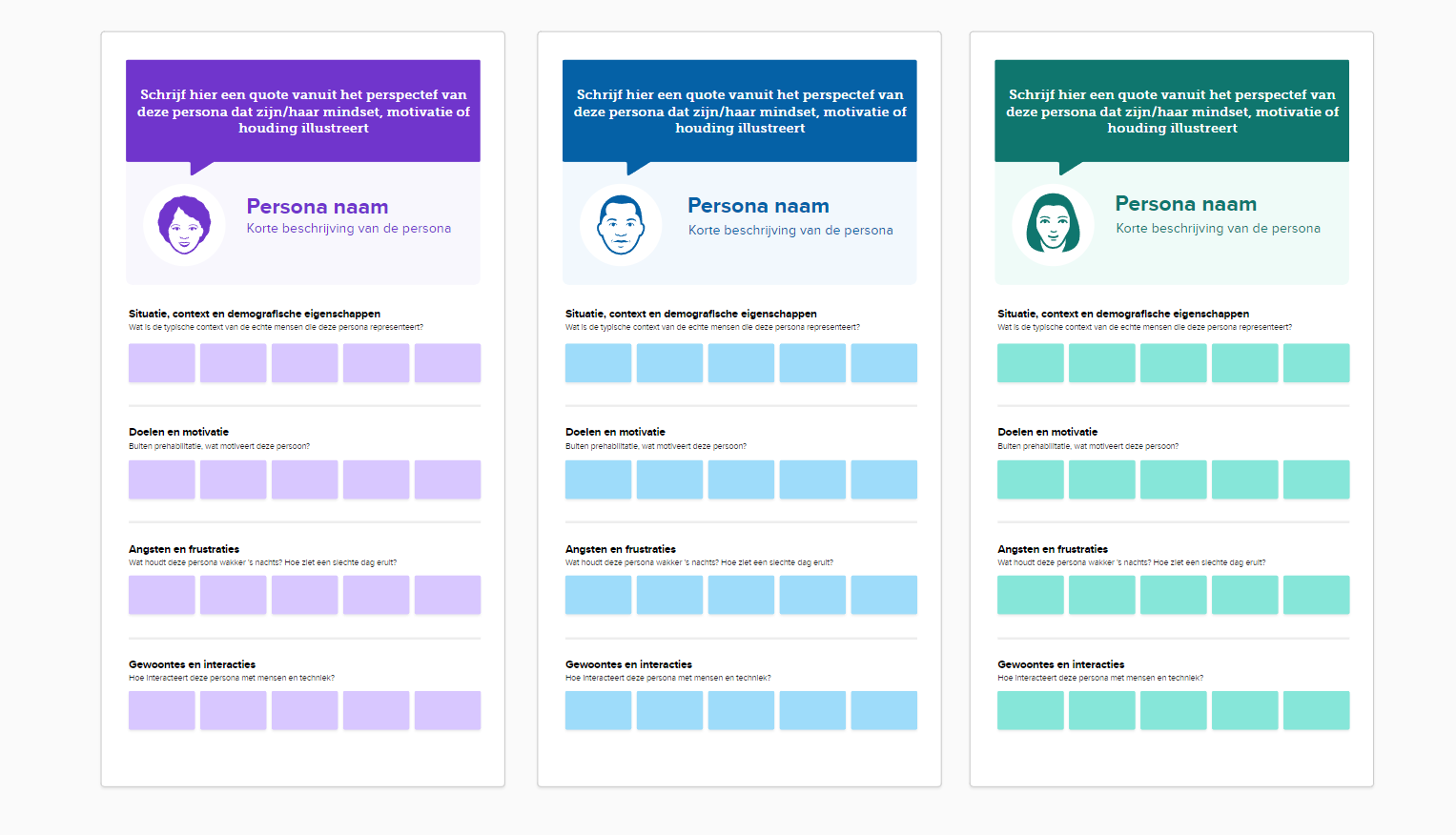

Supplement: Supplementary file 1 — Supplementary material [file mmc1.docx]
